# Bacteremia caused by *Nocardia farcinica*: a case report and literature review

**DOI:** 10.1186/s12879-024-09230-2

**Published:** 2024-04-08

**Authors:** Di Wang, Meng-Ting Hu, Wen-Jing Liu, Ying Zhao, Ying-Chun Xu

**Affiliations:** Department of Clinical Laboratory, State Key Laboratory of Complex Severe and Rare Diseases, Peking Union Medical College Hospital, Chinese Academy of Medical Science and Peking Union Medical College, Beijing, China

**Keywords:** Bacteremia, *Nocardia farcinica*, Adrenal gland abscess, Metagenomic next-generation sequencing

## Abstract

**Background:**

*Nocardia farcinica* is one of the most common *Nocardia* species causing human infections. It is an opportunistic pathogen that often infects people with compromised immune systems. It could invade human body through respiratory tract or skin wounds, cause local infection, and affect other organs via hematogenous dissemination. However, *N. farcinica*-caused bacteremia is uncommon. In this study, we report a case of bacteremia caused by *N. farcinica* in China.

**Case presentation:**

An 80-year-old woman was admitted to Peking Union Medical College Hospital with recurrent fever, right abdominal pain for one and a half month, and right adrenal gland occupation. *N. farcinica* was identified as the causative pathogen using blood culture and plasma metagenomics next-generation sequencing (mNGS). The clinical considerations included bacteremia and adrenal gland abscess caused by *Nocardia* infection. As the patient was allergic to sulfanilamide, imipenem/cilastatin and linezolid were empirically administered. Unfortunately, the patient eventually died less than a month after the initiation of anti-infection treatment.

**Conclusion:**

*N. farcinica* bacteremia is rare and its clinical manifestations are not specific. Its diagnosis depends on etiological examination, which can be confirmed using techniques such as Sanger sequencing and mNGS. In this report, we have reviewed cases of *Nocardia* bloodstream infection reported in the past decade, hoping to improve clinicians’ understanding of *Nocardia* bloodstream infection and help in its early diagnosis and timely treatment.

## Background

*Nocardia* is a genus of aerobic gram-positive actinomycetes. It is typically weak acid-fast positive on staining. It is widely distributed in nature in the soil and sand [1, 2]. There are 251 species of *Nocardia*, of which 54 can cause diseases in humans, according to the List of Prokaryotic names Standing in Nomenclature (http://www.bacterio.net) [3]. Thirteen species, including *Nocardia abscessus*, *Nocardia farcinica*, *Nocardia brasiliensis*, *Nocardia asteroids*, and *Nocardia otitidiscaviarum*, are the most common causes of human infections. *Nocardia* can enter the human body through the respiratory tract or skin wounds, causing local infections and spreading to other organs through blood circulation [4, 5]. Different species exhibit varying antibiotic sensitivities. The microbiological, imaging, and clinical manifestations of *Nocardia* infection have no significant characteristics, its clinical diagnosis rate is low, and it can be easily misdiagnosed or remain undiagnosed [6]. *Nocardial* bacteremia is relatively rare in clinical practice. In this study, we report the case of *Nocardia farcinica* bloodstream infection in a patient admitted to our hospital in 2023; in addition, we reviewed the literature on *Nocardia* bacteremia published in the last 10 years.

## Case presentation

An 80-year-old woman had a history of disseminated nontuberculous mycobacteriosis, cutaneous T-cell lymphoma, lumbar spine fracture, and hypertension. The patient had undergone T6, T12 vertebral compression fractures and right femoral head replacement and was being administered ethambutol, clarithromycin, and valsartan. In January 2023, the patient developed a left shoulder furuncle with a diameter of approximately 1 cm, surrounded by redness and swelling with a small amount of exudation and no obvious fluctuation sensation under the skin. Mupirocin ointment was applied externally, the wound was rinsed with boric acid lotion, and the furuncle improved after approximately 1 month of treatment with oral linezolid. Starting from April 2023, the patient developed recurrent fever with a maximum body temperature of 38 ℃ accompanied by right abdominal pain. Computed tomography (CT) scan in another hospital revealed a mass in the right adrenal gland, indicating the possibility of tumor. After treatment with multiple antibiotics such as ertapenem, voriconazole, moxifloxacin, clarithromycin, and ethambutol, the patient’s symptoms did not improve. The patient was admitted to the emergency Department of Peking Union Medical College Hospital on May 18, 2023. Laboratory tests revealed a white blood cell (WBC) count of 16.2 × 10^9^/L; N% of 93.5%; HGB of 88 g/L; PLT of 241 g/L; and C-reactive protein (CRP), K, Na, and Cr concentrations of 173 mg/L; 3.4 mmol/L; 141 mmol/L; and 40 µmol/L, respectively. The patient was sent for plasma metagenomic next-generation sequencing (mNGS) and blood culture, and anti-infection therapy with 500 mg daptomycin once a day was initiated. As on May 23, 2023, anti-infection treatment was unsuccessful. The patient’s mental state was affected, her appetite gradually decreased, and her condition further worsened. Laboratory tests revealed the following data: WBC, 13.21 × 10^9^/L; RBC, 2.83 × 10^12^/L; HGB, 93 g/L; LY, 1.5%; NET#, 12.44 × 10^9^/L; ALT, 94 U/L; CRP, 164 mg/L; and NT-proBNP, 1409 pg/mL. Plain CT scan revealed a huge, irregular lobed mass with a clear boundary on the right adrenal gland, and adrenal abscess was considered. On May 24, 2023, plasma mNGS identified *Nocardia farcinica*, and the blood aerobic culture was positive after an extended culture of 135 h on the automatic blood culture system. After staining and microscopy, the suspected *Nocardia* finding was immediately reported to the clinic, and further investigations identified the causative pathogen as *No*cardia farcinica. The anaerobic culture vial showed no microbial growth. The inhibition zones of 10 common antibiotics against the *N. farcinica* isolate were detected using an in vitro disk diffusion test (linezolid 32 mm, ciprofloxacin 25 mm, minocycline 22 mm, cefepime 16 mm, cefoxitin 10 mm, amikacin 26 mm, tigecycline 22 mm, imipenem 32 mm, cefotaxime 19 mm, and ceftriaxone 24 mm). The minimum inhibitory concentration (MIC) of Trimethoprim/Sulfamethoxazole (TMP/SMX) detected using the E-test method was 0.25 mg/L. The breakpoints (S ≤ 2/38, *R* ≥ 4/76) for TMP/SMX were determined according to the Clinical and Laboratory Standards Institute guidelines, 3rd Edition (M24). The identified *N. farcinica* isolate is susceptible to TMP/SMX. Since the patient was allergic to sulfanilamide, the clinical adjustment drugs were imipenem, cilastatin sodium, and linezolid, based on antimicrobial susceptibility results. Unfortunately, the patient died less than a month after the initiation of anti-infection therapy.

## Microbiological analysis and molecular examination

**Fig. 1:**
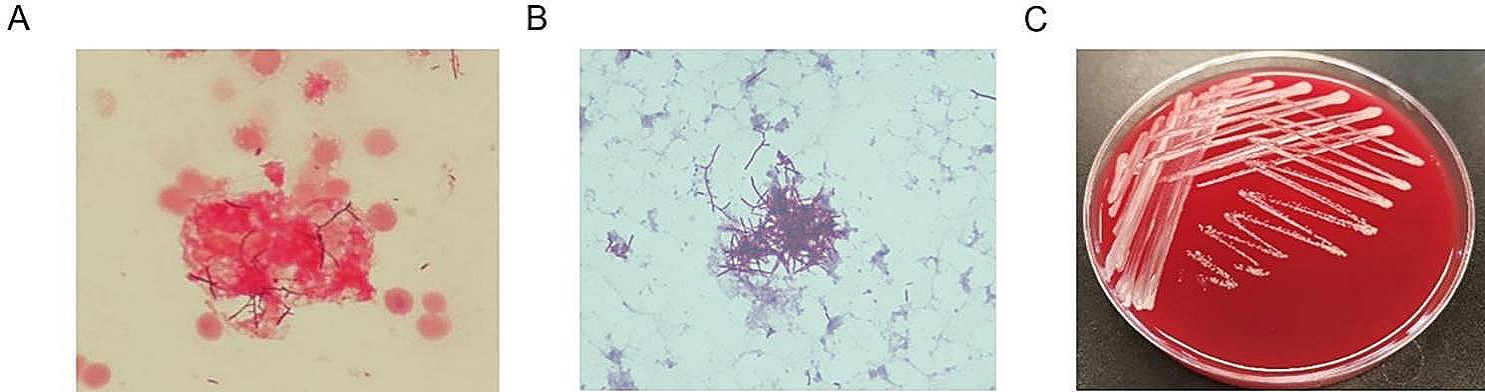
**(A)** Gram staining from positive blood culture (*1000); **(B)** Modified acid-fast staining from positive blood culture (*1000); **(C)** Subcultured colony morphology on blood agar plate

The aerobic blood culture vial was incubated on an automatic blood culture system (BD BACTEC FX, Becton Dickinson) and positive signal was detected after 135 h’s incubation. The vial was subcultured onto a blood agar plate and China Blue agar plate. Microscopic examination of the bacteria showed gram-positive, thin, delicate, branching filamentous organisms (Fig. 1A), and it was positive for modified acid-fast staining (Ziehl-Nielsen stain, but using a weaker decolorizer, 1.0% sulfuric acid) (Fig. 1B). After incubating the subcultured blood agar plate at 35 ℃ for 24 h, wrinkled, dry, round surface colonies were observed (Fig. 1C). The colony was identified as *N. farcinica* using laser-assisted desorption/ionization time-of-flight (score 9.5, with a score of 9.0 or above is considered species level credible) (Autof ms1000, Zhengzhou Autobio Diagnostics).

**Fig. 2:**
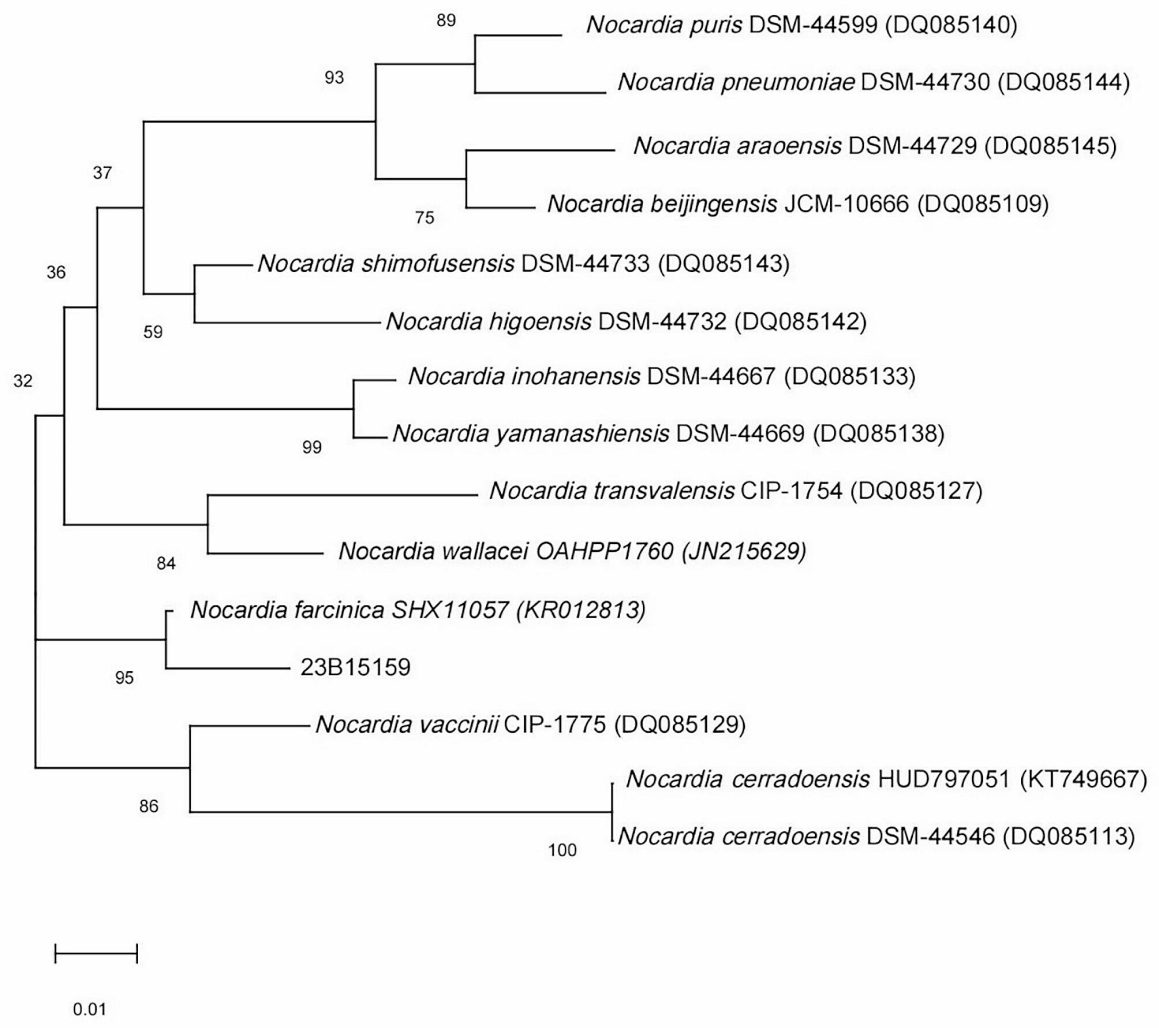
Phylogenetic tree showing the relationship of the blood culture isolate to *N. farcinica* isolates and members of other related genera. The tree was constructed using the neighbor-joining method and bootstrap values calculated from 1000 trees. The accession numbers shown are those in the GenBank database. 23B15159: the clinical *N. farcinica* isolate detected in this study

Several colonies were selected from the subcultured plate to prepare the bacterial suspension, and DNA extraction and rpoB gene sequencing were performed. The following primers were used: forward primer 5′-CGACCACTTCGGCAACCG-3′ and reverse primer 5′-TCGATCGGGCACATCCGG-3′. Species identification was performed by querying the obtained rpoB sequences against those in the GenBank database using the nucleotide Basic Local Alignment Search Tool (BLAST, http://blast.ncbi.nlm.nih.gov). The similarity between the product sequence and rpoB sequence of *N. farcinic*a was 99.72% (https://blast.ncbi.nlm.nih.gov/Blast.cgi), which confirmed the identification of *N. farcinica.* Phylogenetic analysis [7] was performed with the Molecular Evolutionary Genetic Analysis (MEGA) software (version 6.0; http://www.megasoftware.net) using the neighbor-joining method. The phylogenetic tree was built with the clinical isolate 23B15159 and some strains of *N. farcinica* from GenBank and other closely related genera. Phylogenetic tree analysis confirmed that the clinical isolate was *N. farcinica* (Fig. 2). After testing the plasma samples using the standard operating procedures of mNGS laboratory, 400 sequence readings of *N. farcinica* were obtained [8].

## Discussion and conclusions

Nocardiosis is a rare infectious disease caused by the genus *Nocardia*, which often affects multiple organs and can occur in both normal and immunocompromised patients. *Nocardia* bacteremia is rare and occurs in approximately 1.3–7.7% of patients with *Nocardia* infections. The mortality rate of patients with *Nocardia* bacteremia is potentially high, at approximately 50%. The patients do not exhibit typical systemic symptoms, except for local discomfort [2]. We searched relevant literature published worldwide from 2013 to 2023 in PubMed and web of science databases by using “*Nocardia* and blood stream infection” as the keyword. Twenty cases of nocardial bacteremia were retrieved after excluding nonrelevant literature, incomplete clinical data, and possible duplicates (Table 1). In addition to the case reported in our hospital, the clinical characteristics of 21 cases of nocardial bacteremia were summarized and analyzed. The average age of the patients was 61 years and majority of them were male (62%). The most affected areas were the lungs, brain, subcutaneous tissues, and heart. Pre-onset immunocompromised patients, including those with combined immunodeficiency, use of glucocorticoids or immunosuppressants, and hypoproteinemia, accounted for 71.4% of the cases, suggesting that nocardial bacteremia is more likely to occur in middle-aged and older patients with immunocompromised function than in healthy young individuals. The main clinical symptoms of the 21 patients with *Nocardia* bacteremia were fever (71.4%), dyspnea, cough, sputum, headache, and skin abscess; other symptoms varied based on the organ or region infected with *Nocardia*. The main pathological change associated with *Nocardia* infection is suppurative inflammation, which can lead to the formation of abscesses of different sizes [9]. The patient reported in this study had a left shoulder furuncle a few months before presentation. We hypothesized that the *Nocardia* blood infection in this patient may have originated from a skin infection on the left shoulder. CT examination on admission suggested that the patient might have been exhibiting an adrenal abscess, but a puncture could not be made for definite diagnosis. Invasion of the adrenal glands by *Nocardia* is rare [10]. Another possibility is that the left shoulder furuncle may not be the primary site, and *Nocardia* might have entered the body by inhalation and remained dormant, causing a disseminated infection when the host’s immunity reduced [11].

*Nocardia* infection diagnosis can be based on etiological examination (Gram staining, modified acid-fast staining, and culture), infection symptoms, imaging or pathological examination, and mNGS, which has been rapidly developed recently [12]. Generally, it takes 2–7 days for Nocardia to form visible colonies, and it may take several weeks for some species. As a new detection technology, mNGS does not require samples to be cultured and it can directly detect pathogens from various clinical samples such as blood, urine, cerebrospinal fluid, respiratory secretions, and others. Second, mNGS can detect DNA and RNA at the same time, so it can detect various pathogens such as bacteria, fungi, and viruses. Finally, the mNGS assay can provide results in a short period of time and has a high sensitivity to detect pathogens with low abundance. Therefore, if the sample source is complex or difficult to culture, multiple pathogens could be present, and determining the type of pathogen is difficult. Thus, there is a need for timely diagnosis of severe cases, and mNGS has more advantages than traditional culture. However, mNGS is costly and requires specialized equipment and technicians. In practical applications, doctors need to make the best choice based on the patient’s specific situation and clinical needs [13, 14]. In a retrospective analysis of 21 patients, 17 were diagnosed with *Nocardia* bloodstream infection using blood culture and three were diagnosed using blood mNGS. In this case, both blood culture and mNGS were performed, and *N. farcinica* was identified. Among the 21 patients with *Nocardia* bloodstream infection, 18 cases (85.7%) were clearly classified, and *N. farcinica* was the most common pathogen (10 cases), which is consistent with literature reports [15, 16]. *Nocardia* is difficult to diagnose using traditional culture methods. In addition to slow colony growth, patients often have heterogenous infections, and the growth of some other colonies can easily mask that of *Nocardia*. Therefore, for older, immunocompromised patients lacking specific clinical and imaging manifestations, when the treatment effect of broad-spectrum antibiotics is unsatisfactory, and when atypical pathogens, tuberculosis, or fungal infections are suspected, the possibility of *Nocardia* infection should also be considered. In addition to culture, mNGS can be used to identify pathogenic bacteria to avoid delays in diagnosis and treatment.

The first-line treatment for *Nocardia* is sulfonamide. With further research, antibacterial drugs such as third-generation cephalosporins, amikacin, meropenem, imipenem, and linezolid have been recommended for *Nocardia* treatment [17]. Amikacin plays a synergistic role when used in combination with other antibiotics, particularly carbapenems, third-generation cephalosporins, and TMP/SMX. The treatment effect of sulfamethoxazole alone was insufficient, and the combined application of TMP/SMX was stronger than that of each antibiotic alone [18]. Therefore, combination therapy is recommended in most cases of nocardiosis. In the 21 cases of nocardial bacteremia analyzed in this group, 81% involved clinical selection of combined drugs, of which 66.7% achieved a good effect. Treatment is generally recommended for 6–12 months for patients with pulmonary or multifocal (non-central nervous system) nocardiosis and normal immune function. Immunosuppressed patients and those with central nervous system disorders should receive antimicrobial therapy for at least 12 months [19]. Notably, *N. farcinica*, which was the most commonly isolated bacterium in this study, is highly resistant to third-generation cephalosporins, meropenem, ciprofloxacin, and minocycline, and they should be avoided as an empirical treatment [15]. Researchers have analyzed 53 strains of *Nocardia* in seven cities in China, and the results showed that although the resistance rate to sulfonamides varies considerably globally, it remains the first-line treatment for *Nocardia* infection, with sulfamethoxazole as the preferred antibiotic. In addition, amikacin, imipenem, and linezolid can be used as alternative drugs for the initial empirical treatment of nocardiosis in China [20]. After the etiology of our patient was confirmed as an *N. farcinica* infection in our hospital, an E-test antibiotics sensitivity test was performed, and the results indicated that the bacterium was sensitive to TMP-SMX. However, because of the patient’s allergy to sulfonamide, imipenem, cilastatin sodium, and linezolid were clinically selected for infection control. Unfortunately, the patient died less than a month after the inclusion of anti-infection therapy.

Among the patients affected by *Nocardia*, 71.4% had a good prognosis, indicating that nocardial bacteremia is a curable disease; however, six patients succumbed to the disease. One of the possible causes of death was old age with serious underlying diseases and a prolonged history of oral glucocorticoids or immunosuppressants, suggesting that older patients with low immunity should be vigilant about the poor prognosis of *Nocardia* bloodstream infection. Second, the initial unclear diagnosis and delayed treatment are among the factors leading to poor prognosis of nocardial bacteremia. In summary, older patients with underlying diseases and immune dysfunctions are prone to *Nocardia* bloodstream infections. When the clinical use of broad-spectrum antibiotics is not effective and a combination of atypical pathogens, tuberculosis, or fungal infection is suspected, the possibility of *Nocardia* infection should be considered, and blood culture and mNGS should be performed in a timely manner for a clear diagnosis. The first choice of treatment drugs is sulfonamides, with a gradual increase in drug resistance; if necessary, a combination of drugs should be used, and timely drainage should be performed when the abscess spreads to other sites to obtain a good prognosis.

In conclusion, *Nocardia farcinica* bacteremia is rare, and its clinical manifestations lack specificity. Its diagnosis depends on etiological examination, which can also be confirmed using Sanger sequencing, mNGS, and other technologies. The case presented in this report and the review of the cases of *Nocardia* bloodstream infections occurring in the past decade will improve clinicians’ understanding and help in the early diagnosis and timely treatment of *Nocardia* bloodstream infections.

**Table 1:** Review of published *N. farcinica* literature over the past 10 years

Case no.	Age (y), Sex	Symptoms	Underlying disease	Species	Detection method	Detection time (d)	Other dissemination sites	Treatment	Outcome	Reference no.
1	45, M	Fever, cough, chest tightness and fatigue	Uremia, kidney transplant surgery	*N. farcinica*	Plasma NGS	1	Pleura	SMX, MIN	Recovered	[21]
2	91, F	Fever, edema of the right leg	Abdominal aortic aneurysm	*N. farcinica*	Blood culture	6	Lung, lower limbs	TMP-SMX	Died	[22]
3	87, F	Fever	Meningioma	*N. farcinica*	Blood culture	-	Lung	CIP, TMP-SMX	Recovered	[11]
4	77, M	Respiratory distress, blood pressure dropping	Severe pneumonia	*N.cyriacigeorgica*	Blood culture	-	Adrenal gland	LZD, CTM, AK	Recovered	[23]
5	59, M	Paroxysmal cough, phlegm, fever	Bronchiectasis	*N. farcinica*	Plasm mNGS	3	Lung	SMX	Recovered	[24]
6	61, M	Skin redness, swelling, pain, fever	Nephrotic syndrome	*N. farcinica*	Plasm mNGS	1	Lung	TMP-SMX, LZD	Recovered	[25]
7	71, M	Septic shock	Colon adenocarcinoma	*N.brasiliensis*	Blood culture	-	Lung	CRO, LEV	Recovered	[26]
8	49, M	Fever, cough with expectoration, headache and vomiting	Nothing	*N. farcinica*	Blood culture	6	Lung	TMP-SMX	Died	[27]
9	77, M	Difficulty breathing, cough, fever	Chronic obstructive pulmoriary disease	*N. farcinica*	Blood culture	-	Lung	TMP-SMX, IPM, AK	Recovered	[28]
10	53, F	Fatigue, headache, cough, fever	Breast cancer	*Nocardia* species	Blood culture	6	Aortic valve	MPM, AK	Recovered	[29]
11	51, M	Cough, dyspnea, lowgrade fever, generalized weakness and poor appetite	Liver transplant	*N. cyriacigeorgica*	Blood culture	4	Lung	TMP-SMX, IPM	Died	[30]
12	50, M	Watery diarrhea, vomiting, generalized weakness and loss of appetite	Heart transplant	*N. farcinica*	Blood culture	6	Lung	TMP-SMX, MPM, AK	Recovered	[30]
13	67, F	General malaise without high fever and chill	Rheumatoid arthritis	*Nocardia brasiliensis*	Blood culture	5	Lung, haunch	MPM, TMP-SMX, AK	Recovered	[31]
14	56, M	Fatigue, anorexia, severe weight loss, dyspnea, hemoptysis	Psoriasis	*Nocardia*	Blood culture	-	Lung, kidneys, brain, skin	LZD, AK, TMP-SMX	Recovered	[32]
15	66, M	Fever and vomiting	Diffuse large B-cell lymphoma	*N. otitidiscaviarum*	Blood culture	5	Lung, brain	TMP-SMX, LZD	Recovered	[33]
16	58, M	Nasal congestion and dry cough	Autologous peripheral blood stem cell transplantation	*Nocardia nova*	Blood culture	-	Lung	TMP-SMX, IPM	Died	[34]
17	58, F	Shortness of breath, fever	Chronic obstructive pulmoriary disease	*N. farcinica*	Blood culture	-	Heart	MPM, AK	Recovered	[35]
18	51, M	Septic shock	Acute myeloid leukemia	*Nocardia veterana*	Blood culture	-	Lung	TMP-SMX	Died	[36]
19	59, F	Headache, fever, cough, difficulty breathing	Renal transplantation	*N. cerradoensis*	Blood culture	-	Lung, brain	MPM, AK, TMP-SMX	Recovered	[37]
20	18, F	Fever, cough and chest pain	Sickle Cell Anemia	*Nocardia*	Blood culture	1	Lung, kidney	TMP-SMX, AK, CRO	Recovered	[38]

TMP/SMX, Trimethoprim/ Sulfamethoxazole; MIN, Minocycline; CIP, Ciprofloxacin; LZD, Linezolid; CTM, Cefotaxime; AK, Amikacin; CRO, Ceftriaxone; LEV, Levofloxacin; IPM, Imipenem; MPM, Meropenem; “-”: This information is unknown

## Data Availability

The datasets used and/or analyzed during the current study are available from the corresponding author on reasonable request.

## Abbreviations

CT: Computed Tomography
TMP/SMX: Trimethoprim/Sulfamethoxazole
mNGS: metagenomic next-generation sequencing
WBC: white blood cell
